# Multidisciplinary expert group: communication measures to increase vaccine compliance in adults

**DOI:** 10.1007/s10354-022-00946-x

**Published:** 2022-07-06

**Authors:** Matthias R. Hastall, Isabell Koinig, Ursula Kunze, Oliver Meixner, Katharina Sachse, Reinhard Würzner

**Affiliations:** 1 Faculty of Rehabilitation Sciences, Technical University of Dortmund, Dortmund, Germany; 2 Department of Media and Communication Studies, University of Klagenfurt, Klagenfurt, Austria; 3 Center for Public Health, Medical University of Vienna, Vienna, Austria; 4 Institute of Marketing and Innovation, University of Natural Resources and Life Sciences, Vienna, Austria; 5 Business psychology, FOM University of Applied Sciences, Berlin, Germany; 6 Institute for Hygiene and Medical Microbiology, Medical University of Innsbruck, Innsbruck, Austria

**Keywords:** Infectious diseases, Vaccine preventable diseases, Vaccination recommendations, Online communication, Targeting and tailoring strategies

## Abstract

The WHO categorized vaccine hesitancy as one of the greatest threats to global health worldwide. Vaccination of elderly persons is of increasing relevance, given that they represent a growing segment in the population and considering the burden diseases pose to them. Many factors leading to vaccine hesitancy are related to inadequate communication. In the present report, experts from various academic fields present recommendations to support communication strategies that may help to initiate targeted communication measures to enhance vaccination compliance in adults.

In 2019, before we knew anything at all about the Coronavirus pandemic (COVID-19 pandemic), the World Health Organisation (WHO) called vaccine hesitancy the greatest threat to global health amongst the most important health threats worldwide [1]. The discussion in favor of or against a potential COVID-19 vaccination is currently ongoing and indirectly influences other vaccinations as well.

In the present report, a multidisciplinary group of experts from Austria and Germany (working in the fields of strategic health communication, marketing and innovation, psychology and hygiene, public health, and medical microbiology) discuss several aspects of vaccination and, specifically, how vaccination compliance among adults could be increased. As communication is determined to be a key factor in supporting higher vaccination compliance, recommendations are presented to support targeted communication measures.

## Introduction

Vaccinations are one of the greatest success stories of modern medicine and have proven to be a successful instrument to combat life-threatening infectious diseases in children and adults. Especially vaccination of elderly persons is of increasing relevance, given that they represent a growing segment in the overall population and considering the great burden diseases pose to them, e.g., as in the case of vaccine-preventable diseases. By 2050, 30% of the European population will be older than 60 years. Despite the major relevance of vaccinations to the elderly, we lack any international consensus on recommendations for vaccination in adults [2] and immunization coverage for some vaccinations is far from adequate [3].

National vaccine recommendations for adults do exist in some countries, including Austria and Germany, but in fact, just a part of these recommendations is currently implemented. There are multiple reasons for this, for example low problem awareness among health care workers or lack of reimbursement of vaccine costs in Austria.

Whether a person gets vaccinated depends on a variety of factors. The WHO has focused on individual as well as contextual determinants of vaccination behavior. Individual determinants include risk perception, trust/distrust, and perceived obstacles. Contextual determinants encompass cultural factors, social norms, socioeconomic status, education levels, and the structure of the respective health care system [3].

In most European countries, as well as in other countries throughout the world, we are observing the rise of several groups that are hostile towards vaccinations, delay their administration, or entirely reject their use. This phenomenon is referred to as vaccine hesitancy [4]. Vaccine hesitancy is now a problem that affects 90% of all countries throughout the world [5].

The WHO defines vaccine hesitancy as a behavior that is influenced by a variety of factors: trust (in vaccines or their suppliers), self-satisfaction (the need for vaccines is not perceived), and convenience (access to vaccination). Persons who hesitate to get vaccinated constitute an extremely heterogeneous group and react to vaccines with skepticism or hesitancy of varying degrees [6].

Of equal importance is the lack of information or insufficient information on the part of doctors [7] and the existence of alternative lifestyles [8], as we know from the multitude of opinions about healthy food.

Several explanatory models of vaccine hesitancy have been developed over the years. The most well-known models are the 5C model [9], the 5A model [10], the CMO‑B model [3], and the SAGE model [11].

According to these models, many but not all factors leading to vaccine hesitancy are related to communication. In most instances, poor or inadequate communication is likely to negatively influence vaccination coverage and can enhance vaccine hesitancy [11].

## The importance of optimized vaccination information

The environment in which decisions concerning vaccination are made is of significant importance [12]. If the environment provides a large body of scientifically confirmed data, it is called a safe environment. However, if extensive false information is available in the environment, it is referred to as a polluted environment. This includes so-called scientific skepticism or the impeachment of scientific data [13] and denialism, i.e., the validity of indisputable scientific data is denied [14]. In such an environment, it is difficult to convince individuals by presenting reliable scientific information.

Persons who act in accordance with national recommendations and receive vaccinations and those opposing vaccinations do not interact respectfully with each other; this leads to an escalation of the situation. Each party uses information and arguments that lead to their desired conclusion. Some experts believe that the aims of the two parties are not essentially different, as both are concerned with safety and health. Therefore, reducing the escalation would be a first step towards improved communication.

Persons who oppose vaccinations must be taken seriously and addressed appropriately. Only then will it be possible to convince them of the importance of vaccinations. In such communicative encounters, the main focus should be on the way we create messages, e.g., the emotionalization of messages [15], the need to reduce message complexity [16], and how message arguments are presented (e.g., in a dual-mode presentation) [17]. On the other hand, the consideration of media channels and social media communication opportunities [18], as well as specific targeting and tailoring of messages to the needs and requirements of the target group [19] is deemed important (Table 1). The aims of such efforts are to enhance the demand for vaccinations [3], build up knowledge on the subject, and to avoid false information and its negative consequences [20].

**Table 1:** How messages can be developed to promote more effective vaccine communication

Effective messages for vaccine communication
Use storytelling
Use emotional messages
Show vulnerability and self-efficacy
Use nudging message options
Use testimonials (by prominent endorsers, physicians, experts, etc.)
Use vivid language dual-mode text/picture
Correct false information
Enhance comprehensibility

**Table 2:** How online communication can be used to enhance vaccine communication

Effective online communication
Defines target groups and selects online media accordingly
Uses targeting and tailoring (platform and content)
Creates knowledge platforms in common language
Creates barrier-free information
Utilizes multiplicators
Uses search engine optimization (SEO)
Enhances awareness of trustworthy websites
Promotes information exchange

## How to develop messages for the general population

The emotionalization of messages and storytelling are very important tools for delivering messages to the general population [21]. Case studies or testimonial statements by experts (e.g., doctors) or persons of the target group (community members) can enhance the recipient’s identification with the message [15]. Positive effects include intensified attention towards and acceptance of messages [22] as well as enhanced credibility [23]. Successful examples are the campaign “Widowed by Influenza” by the Health Ministry of North Rhine-Westphalia in 2008 (personal communication) and the website www.shotbyshot.org, which features an emotional presentation of the personal fates and consequences for persons affected by vaccine-preventable diseases.

When designing messages for a specific target group, messages should be based on both theory and practice [24]. Dual-mode presentation should be used to support arguments by integrating illustrations and visuals to reduce complexity and to facilitate comprehension and processing [25]. If the information and arguments are presented in dual-mode, low-functional literacy—i.e., individual’s ability to read—can be overcome [26].

Equally important is the combination of emotionally charged messages with easy public access to vaccinations. One example could be the on-site offer of information (i.e., the need for a vaccination and the potential effect of non-vaccination) for hospital staff, combined with the immediate opportunity of vaccination on site, like in a hospital cafeteria.

The significant role of health care workers in vaccination has been mentioned in several studies [7]. By vaccinating themselves, persons in health care professions can protect their patients from diseases preventable by vaccination. By doing so, they could also serve as role models.

## Effective use of online communication (Table 2)

The greatest backlog exists in the usage of social media, as social networking sites are utilized more effectively by persons opposed to vaccinations. The interactive potential of websites and social media is currently utilized by pro-vaccination groups to a very limited extent [27]. Forums should not be surrendered to anti-vaccinationists but utilized by vaccination experts as well, as the reticence of pro-vaccinationists has indirectly supported the anti-vaccination information presented in these forums.

An important consideration is the selection of appropriate media channels for different age and target groups. Specific targeting and tailoring strategies are used to design messages for a specific target group and personalize the communication [19]. One approach is the use of educated multiplicators who could drive the involvement and initiate discussions with their followers to promote an information exchange [18]. Vaccination experts could host knowledge platforms with trustworthy information, correct false information, and even utilize search engine optimization for delivering their messages to the target group. Effective pro-vaccination messages in targeted online communication channels could even serve as a vaccination knowledge base and inform potential vaccine-skeptical adults outside the traditional healthcare information settings.
